# Schur-convexity, Schur-geometric and Schur-harmonic convexity for a composite function of complete symmetric function

**DOI:** 10.1186/s40064-016-1940-z

**Published:** 2016-03-08

**Authors:** Huan-Nan Shi, Jing Zhang, Qing-Hua Ma

**Affiliations:** Department of Electronic Information, Teacher’s College, Beijing Union University, Beijing, 100011 People’s Republic of China; Basic Courses Department, Beijing Union University, Beijing, 100101 People’s Republic of China; College of Applied Arts and Science, Beijing Union University, Beijing, 100083 People’s Republic of China

**Keywords:** Schur-convexity, Schur-geometric convexity, Schur-harmonic convexity, Complete symmetric function, Primary 05E05, 26B25

## Abstract

In this paper, using the properties of Schur-convex function, Schur-geometrically convex function and Schur-harmonically convex function, we provide much simpler proofs of the Schur-convexity, Schur-geometric convexity and Schur-harmonic convexity for a composite function of the complete symmetric function.

## Background

Throughout the article, $\ {\mathbb {R}}$ denotes the set of real numbers, $\varvec{x} = (x_1, x_2, \ldots , x_n)$ denotes *n*-tuple (*n*-dimensional real vectors), the set of vectors can be written as

$$\begin{aligned}&{\mathbb {R}}^n = \left\{ {\varvec{x}=(x_1, x_2, \cdots , x_n): x_i \in {\mathbb {R}}, i = 1,2,\ldots ,n} \right\} ,\\&{\mathbb {R}}^{n}_{+}=\{\varvec{x}=(x_{1},x_{2},\ldots ,x_{n}): x_{i}>0, i=1,2,\ldots ,n\},\\&{\mathbb {R}}^{n}_{-}=\{\varvec{x}=(x_{1},x_{2},\ldots ,x_{n}): x_{i}<0, i=1,2,\ldots ,n\}. \end{aligned}$$

In particular, the notations ${\mathbb {R}}$ and ${\mathbb {R}}_{+}$ denote ${\mathbb {R}}^{1}$ and ${\mathbb {R}}^{1}_{+}$, respectively.

The following complete symmetric function is an important class of symmetric functions.

For $\varvec{x}=(x_1,x_2,\ldots ,x_n) \in {\mathbb {R}}^n$, the complete symmetric function $c_n(\varvec{x},r)$ is defined as

1 $$\begin{aligned} c_n(\varvec{x},r)=\sum \limits _{i_1+ i_2+\cdots +i_n=r}x_1^{i_1}x_2^{i_2}\cdots x_n^{i_n}, \end{aligned}$$

where $$c_0(\varvec{x},r)=1, \,r\in \{1,2,\ldots , n \},\,$$$$i_1,i_2,\ldots , i_n$$ are non-negative integers.

It has been investigated by many mathematicians and there are many interesting results in the literature.

Guan (2006) discussed the Schur-convexity of $c_n(\varvec{x},r)$ and proved that $c_n(\varvec{x},r)$ is increasing and Schur-convex on ${\mathbb {R}}^n_+$. Subsequently, Chu et al. (2011) proved that $c_n(\varvec{x},r)$ is Schur-geometrically convex and harmonically convex on ${\mathbb {R}}^n_+$.

Recently, Sun et al. (2014) studied the Schur-convexity, Schur-geometric convexity and Schur-harmonic convexity of the following composite function of $c_n(\varvec{x},r)$

2 $$\begin{aligned} F_n(\varvec{x},r)=\sum \limits _{i_1+ i_2+\cdots +i_n=r}\left( \frac{x_1}{1-x_1}\right) ^{i_1}\left( \frac{x_2}{1-x_2}\right) ^{i_2}\cdots \left( \frac{x_n}{1-x_n}\right) ^{i_n}. \end{aligned}$$

Using the Lemma 1, Lemma 2 and Lemma 3 in second section, they proved as follows: Theorem A, Theorem B and Theorem C, respectively.

### **Theorem A**

*For*$$\varvec{x}=(x_1,x_2,\ldots ,x_n) \in [0,1)^n\cup (1,+\infty )^n$$*and*$$r \in {\mathbb {N}}$$*,*

- (i) $$F_n(\varvec{x},r)$$*is increasing in*$$x_{i}$$*for alli*$$\in$$$$\{1, 2, \ldots , n\}$$*and Schur-convex on*$$[0, 1)^n$$*for eachrfixed;*
- (ii) *ifris even integer (or odd integer, respectively), then*$F_n(\varvec{x},r)$*is Schur-convex (or Schur-concave, respectively) on*$(1,+\infty )^n$*, and it is decreasing (or increasing, respectively) in*$x_{i}$*for all*$i \in \{1,2,\ldots ,n\}$.

### **Theorem B**

*For*$$\varvec{x}=(x_1,x_2,\ldots ,x_n) \in [0,1)^n\cup (1,+\infty )^n$$*and*$$r \in \mathbb {N}$$*,*

- (*i*) $$F_n(\varvec{x},r)$$*is Schur-geometrically convex on*$$[0, 1)^n$$*;*
- (*ii*) *ifris even integer (or odd integer, respectively), then*$F_n(\varvec{x},r)$*is Schur-geometrically convex (or Schur-geometrically concave, respectively) on*$(1,+\infty )^n$.

### **Theorem C**

*For*$$\varvec{x}=(x_1,x_2,\ldots ,x_n) \in [0,1)^n\cup (1,+\infty )^n$$*and*$$r \in \mathbb {N}$$*,*

- (i) $$F_n(\varvec{x},r)$$*is Schur-harmonically convex on*$$[0, 1)^n$$*;*
- (ii) *ifris even integer (or odd integer, respectively), then*$F_n(\varvec{x},r)$*is Schur-harmonically convex (or Schur-harmonically concave, respectively) on*$(1,+\infty )^n$.

In this paper, using the properties of Schur-convex function, Schur-geometrically convex function and Schur-harmonically convex function, we will provide much simpler proofs of the above results.

## Definitions and lemmas

For convenience, we recall some definitions as follows.

### **Definition 1**

Let $$\varvec{x} = ( x_{1},x_{2},\ldots , x_{n })$$ and $$\varvec{y} = ( y_{1},y_{2},\ldots , y_{n }) \in {\mathbb {R}}^{n}$$.

- (i) $\varvec{x}\ge \varvec{y}$ means $x_{i} \ge y_{i}$ for all $i=1, 2, \ldots , n$.
- (ii) Let $\Omega \subset {\mathbb {R}} ^{n}$, $\varphi$: $\Omega \rightarrow {\mathbb {R}}$ is said to be increasing if $\varvec{x} \ge \varvec{y}$ implies $\varphi {(\varvec{x})} \ge \varphi {(\varvec{y})}$. $\varphi$ is said to be decreasing if and only if $-\varphi$ is increasing.

### **Definition 2**

Let $$\varvec{x} = ( x_{1},x_{2},\ldots , x_{n })$$ and $$\varvec{y} = ( y_{1},y_{2},\ldots , y_{n }) \in {\mathbb {R}}^{n}$$.

(i): $\varvec{x}$ is said to be majorized by $\varvec{y}$ (in symbols $\varvec{x} \prec \varvec{y}$) if $\sum _{i = 1}^k x_{[i]} \le \sum _{i = 1}^k y_{[i]}$ for $k = 1,2,\ldots ,n - 1$ and $\sum _{i = 1}^n x_i = \sum _{i = 1}^n y_i$, where $x_{[1]}\ge x_{[2]}\ge \cdots \ge x_{[n]}$ and $y_{[1]}\ge y_{[2]}\ge \cdots \ge y_{[n]}$ are rearrangements of $\varvec{x}$ and $\varvec{y}$ in a descending order.
(ii): Let $$\Omega \subset {\mathbb {R}}^{n}$$, $$\varphi$$: $$\Omega \rightarrow {\mathbb {R}}$$ is said to be a Schur-convex function on $$\Omega$$ if $$\varvec{x} \prec \varvec{y}$$ on $$\Omega$$ implies $$\varphi \left( \varvec{x} \right) \le$$$$\varphi \left( \varvec{y} \right) .$$ The function $$\varphi$$ is said to be Schur-concave on $$\Omega$$ if and only if $$- \varphi$$ is a Schur-convex function on $$\Omega$$.

### **Definition 3**

Let $$\varvec{x} = ( x_{1},x_{2},\ldots , x_{n })$$ and $$\varvec{y} = ( y_{1},y_{2},\ldots , y_{n }) \in {\mathbb {R}}^{n}$$.

- (i) $\Omega \subset {\mathbb {R}}^{n}$ is said to be a convex set if $\varvec{x},\varvec{y}\in \Omega , 0 \le \alpha \le 1$, implies $\alpha \varvec{x}+(1-\alpha )\varvec{y}=\left( \alpha x_1+(1-\alpha )y_1,\alpha x_2+(1-\alpha )y_2,\ldots ,\alpha x_n+(1-\alpha )y_n\right) \in \Omega$.
- (ii) Let $\Omega \subset {\mathbb {R}}^{n}$ be a convex set. A function $\varphi$: $\Omega \rightarrow {\mathbb {R}}$ is said to be convex on $\Omega$ if
  $$\begin{aligned} \varphi \left( \alpha \varvec{x}+(1-\alpha )\varvec{y}\right) \le \alpha \varphi (\varvec{x})+(1-\alpha )\varphi (\varvec{y}) \end{aligned}$$
  for all $\varvec{x},\varvec{y}\in \Omega$, and all $\alpha \in [0,1]$. The function $\varphi$ is said to be concave on $\Omega$ if and only if $- \varphi$ is a convex function on $\Omega$.

### **Definition 4**

- (i) A set $\Omega \subset {\mathbb {R}}^{n}$ is called symmetric, if $\varvec{x}\in \Omega$ implies $\varvec{x}P \in \Omega$ for every $n\times n$ permutation matrix *P*.
- (ii) A function $\varphi : \Omega \rightarrow {\mathbb {R}}$ is called symmetric if for every permutation matrix *P*, $\varphi (\varvec{x}P) = \varphi (\varvec{x})$ for all $\varvec{x} \in \Omega$.

### **Lemma 1**

(Schur-convex function decision theorem) (Marshall et al. 2011, p. 84) *Let*$\Omega \subset {\mathbb {R}} ^n$*be symmetric convex set with nonempty interior.*$\Omega ^0$*is the interior of*$\Omega$*. The function*$\varphi :\Omega \rightarrow {\mathbb {R}}$*is continuous on*$\Omega$*and continuously differentiable on*$\Omega ^0$*. Then*$\varphi$*is a*$Schur-convex\,(or\,Schur-concave,\,respectively)\,function$*if and only if*$\varphi$*is symmetric on*$\Omega$*and*

3 $$\begin{aligned} \left( x_1 - x_2 \right) \left( \frac{\partial \varphi }{\partial x_1} - \frac{\partial \varphi }{\partial x_2 } \right) \ge 0 \quad (or\le 0,\,respectively) \end{aligned}$$

*holds for any*$\varvec{x} \in \Omega ^0$.

The first systematical study of the functions preserving the ordering of majorization was made by Issai Schur in 1923. In Schur’s honor, such functions are said to be “Schur-convex”. It has many important applications in analytic inequalities, combinatorial optimization, quantum physics, information theory, and other related fields. See Marshall et al. (2011), Rovenţa (2010), Čuljak et al. (2011), Zhang and Shi (2014).

### **Definition 5**

Let $$\Omega \subset {\mathbb {R}}_{+}^{n}$$, $$\varvec{x} = ( x_{1},x_{2},\ldots , x_{n })$$ and $$\varvec{y} = ( y_{1},y_{2},\ldots , y_{n }) \in {\mathbb {R}}_{+}^{n}$$.

(i): (Zhang 2004, p. 64) $\Omega$ is called a geometrically convex set if $(x_{1}^{\alpha }y_{1}^{\beta },x_{2}^{\alpha }y_{2}^{\beta },\ldots ,x_{n}^{\alpha }y_{n}^{\beta }) \in \Omega$ for all $\varvec{x}$, $\varvec{y} \in \Omega$ and $\alpha$, $\beta \in [0, 1]$ such that $\alpha +\beta =1$.
(ii): (Zhang 2004, p. 107) The function $\varphi$: $\Omega \rightarrow {\mathbb {R}}_+$ is said to be a Schur-geometrically convex function on $\Omega$, for any $\varvec{x}, \varvec{y} \in \Omega$, if
  $$\begin{aligned} (\log x_{1},\log x_{2},\ldots ,\log x_{n}) \prec (\log y_{1},\log y_{2},\ldots , \log y_{n}) \end{aligned}$$
  implies $\varphi \left( \varvec{x} \right) \le \varphi \left( \varvec{y} \right)$. The function $\varphi$ is said to be a Schur-geometrically concave function on $\Omega$ if and only if $- \varphi$ is a Schur-geometrically convex function on $\Omega$.

By Definition 5, the following is obvious.

### **Proposition 1**

*Let*$\Omega \subset {\mathbb {R}}_{+}^n$*, and let*

$$\begin{aligned} \log \Omega = \{( \log x_{1},\log x_{2},\ldots , \log x_{n }) : ( x_{1},x_{2},\ldots , x_{n }) \in \Omega \}. \end{aligned}$$

*Then*$\varphi :\Omega \rightarrow {\mathbb {R}}_+$*is a Schur-geometrically convex (or Schur-geometrically concave, respectively) function on*$\Omega$*if and only if*$\varphi (e^{x_1},e^{x_2},\ldots ,e^{x_n})$*is a Schur-convex (or Schur-concave, respectively) function on*$\log \Omega$.

### **Lemma 2**

(Schur-geometrically convex function decision theorem) (Zhang 2004, p.108) *Let*$\Omega \subset {\mathbb {R}}_ {+} ^n$*be a symmetric and geometrically convex set with a nonempty interior*$\Omega ^0$*. Let*$\varphi :\Omega \rightarrow {\mathbb {R}}_+$*be continuous on*$\Omega$*and differentiable in*$\Omega ^0$*. If*$\varphi$*is symmetric on*$\Omega$*and*

4 $$\begin{aligned} \left( {\log x_1 -\log x_2 } \right) \left( {x_1 \frac{\partial \varphi }{\partial x_1 } - x_2 \frac{\partial \varphi }{\partial x_2 }} \right) \ge 0 \quad (or \le 0, respectively) \end{aligned}$$

*holds for any*$\varvec{x} = \left( {x_1, x_2, \ldots ,x_n } \right) \in \Omega ^0$*, then*$\varphi$*is a Schur-geometrically convex (or Schur-geometrically concave, respectively) function.*

The Schur-geometric convexity was proposed by Zhang (2004), and was investigated by Chu et al. (2008), Guan (2007), Sun et al. (2009), and so on. We also note that some authors use the term “Schur multiplicative convexity”.

In 2009, Chu (Chu et al. (2011), Chu and Sun (2010), Chu and Lv (2009)) introduced the notion of Schur-harmonically convex function.

### **Definition 6**

Chu and Sun (2010) Let $\Omega \subset {\mathbb {R}}_{+}^{n}$, $\varvec{x} = ( x_{1},x_{2},\ldots , x_{n })$ and $\varvec{y} = ( y_{1},y_{2},\ldots , y_{n }) \in {\mathbb {R}}_{+}^{n}$.

- (i) A set $\Omega$ is said to be harmonically convex if $( \frac{2 x_1 y_1}{x_1+y_1}, \frac{2 x_2 y_2}{x_2+y_2}, \ldots , \frac{2 x_n y_n}{x_n+y_n}) \in \Omega$ for every ${\varvec{x},\varvec{y}}\in \Omega$.
- (ii) A function $\varphi :\Omega \rightarrow {\mathbb {R}}_+$ is said to be Schur-harmonically convex on $\Omega$, for any $\varvec{x}, \varvec{y} \in \Omega$, if $( \frac{1}{x_{1}}, \frac{1}{x_{2}}, \ldots , \frac{1}{x_{n}}) \prec ( \frac{1}{y_{1}}, \frac{1}{y_{2}}, \ldots , \frac{1}{y_{n}})$ implies $\varphi ({\varvec{x}}) \le \varphi ({\varvec{y}})$. A function $\varphi$ is said to be a Schur-harmonically concave function on $\Omega$ if and only if $-\varphi$ is a Schur-harmonically convex function on $\Omega$.

By Definition6, the following is obvious.

### **Proposition 2**

*Let*$\Omega \subset {\mathbb {R}}_{+}^n$*be a set, and let*$\frac{1}{\Omega } = \{( \frac{1}{x_1}, \frac{1}{x_2}, \ldots , \frac{1}{x_n}) :( x_{1},x_{2},\ldots , x_{n }) \in \Omega \}$*. Then*$\varphi :\Omega \rightarrow {\mathbb {R}}_+$*is a Schur-harmonically convex (or Schur-harmonically concave, respectively) function on*$\Omega$*if and only if*$\varphi \left ( \frac{1}{x_1}, \frac{1}{x_2},\ldots ,\frac{1}{x_n}\right)$*is a Schur-convex (or Schur-concave, respectively) function on*$\frac{1}{\Omega }$.

### **Lemma 3**

(Schur-harmonically convex function decision theorem) (Chu and Sun 2010) *Let*$\Omega \subset {\mathbb {R}}_+^n$*be a symmetric and harmonically convex set with inner points and let*$\varphi :\Omega \rightarrow {\mathbb {R}}_+$*be a continuous symmetric function which is differentiable on*$\Omega ^0$*. Then*$\varphi$*is Schur-harmonically convex (or Schur-harmonically concave, respectively) on*$\Omega$*if and only if*

5 $$\begin{aligned} (x_1-x_2)\biggl (x_1^2 \frac{\partial \varphi }{\partial x_1} -x_2^2 \frac{\partial \varphi }{\partial x_2}\biggr ) \ge 0\quad (or \le 0, respectively),\quad \varvec{x} \in \Omega ^0. \end{aligned}$$

### **Lemma 4**

*Ifris even integer (or odd integer, respectively), then*$c_n(\varvec{x},r)$*is decreasing and Schur-convex (or increasing and Schur-concave, respectively) on*${\mathbb {R}}^{n}_{-}$*.*

### *Proof*

Notice that

$$\begin{aligned}&c_n(\varvec{-x},r)\\&\quad =\sum \limits _{i_1+i_2+\cdots +i_n=r}(-x_1)^{i_1}(-x_2)^{i_2}\cdots (-x_n)^{i_n}\\&\quad =(-1)^{i_1+i_2+\cdots +i_n}\sum \limits _{i_1+i_2+\cdots +i_n=r}x_1^{i_1}x_2^{i_2}\cdots x_n^{i_n}\\&\quad =(-1)^rc_n(\varvec{x},r), \end{aligned}$$

i.e.

$$\begin{aligned} c_n(\varvec{-x},r)=(-1)^rc_n(\varvec{x},r). \end{aligned}$$

If *r* is even integer, then $c_n(\varvec{x},r)=c_n(-\varvec{x},r)$. For $\varvec{x},\varvec{y}\in {\mathbb {R}}^{n}_{-}$, if $\varvec{x}\prec \varvec{y}$, then $-\varvec{x}\prec -\varvec{y}$ and $-\varvec{x},-\varvec{y} \in {\mathbb {R}}^{n}_{+}$, but $c_n(\varvec{x},r)$ is Schur-convex in ${\mathbb {R}}^n_+$, so that $c_n(-\varvec{x},r)\le c_n(-\varvec{y},r)$, i.e. $c_n(\varvec{x},r)\le c_n(\varvec{y},r)$, this shows that $c_n(\varvec{x},r)$ is Schur-convex in ${\mathbb {R}}^{n}_{-}$. If $\varvec{x}\le \varvec{y}$, then $-\varvec{x}\ge -\varvec{y}$, but $c_n(\varvec{x},r)$ is increasing in ${\mathbb {R}}^n_+$, so that $c_n(-\varvec{x},r)\ge c_n(-\varvec{y},r)$, i.e. $c_n(\varvec{x},r)\ge c_n(\varvec{y},r)$, this shows that $c_n(\varvec{x},r)$ is decreasing in ${\mathbb {R}}^{n}_{-}$.

If *r* is odd integer, then $c_n(\varvec{x},r)=-c_n(-\varvec{x},r)$. For $\varvec{x},\varvec{y}\in {\mathbb {R}}^{n}_{-}$, if $\varvec{x}\prec \varvec{y}$, then $-\varvec{x}\prec -\varvec{y}$ and $-\varvec{x},-\varvec{y} \in {\mathbb {R}}^{n}_{+}$, but $c_n(\varvec{x},r)$ is Schur-convex in ${\mathbb {R}}^n_+$, so that $c_n(-\varvec{x},r)\le c_n(-\varvec{y},r)$, i.e. $c_n(\varvec{x},r)\ge c_n(\varvec{y},r)$, this shows that $c_n(\varvec{x},r)$ is Schur-concave in ${\mathbb {R}}^{n}_{-}$. If $\varvec{x}\le \varvec{y}$, then $-\varvec{x}\ge -\varvec{y}$, but $c_n(\varvec{x},r)$ is increasing in ${\mathbb {R}}^n_+$, so that $c_n(-\varvec{x},r)\ge c_n(-\varvec{y},r)$, i.e. $c_n(\varvec{x},r)\le c_n(\varvec{y},r)$, this shows that $c_n(\varvec{x},r)$ is increasing in ${\mathbb {R}}^{n}_{-}$. $\square$

### **Lemma 5**

(Marshall et al. 2011, p. 91; Wang 1990, p. 64–65) *Let the set *${\mathbb {A}}, {\mathbb {B}}\subset {\mathbb {R}}$*,*$\varphi :{\mathbb {B}}^n\rightarrow {\mathbb {R}}$*,*$f:{\mathbb {A}}\rightarrow {\mathbb {B}}$*and*$\psi (x_1, x_2, \ldots , x_n) = \varphi (f(x_1),f(x_2), \ldots , f(x_n)):{\mathbb {A}}^n\rightarrow {\mathbb {R}}$*.*

- (i) *If*$\varphi$*is increasing and Schur-convex andfis increasing and convex, then*$\psi$*is increasing and Schur-convex.*
- (ii) *If*$\varphi$*is decreasing and Schur-convex andfis increasing and concave, then*$\psi$*is decreasing and Schur-convex*.
- (iii) *If*$\varphi$*is increasing and Schur-concave andfis increasing and concave, then*$\psi$*is increasing and Schur-concave.*
- (iv) *If*$\varphi$*is decreasing and Schur-convex andfis decreasing and concave, then*$\psi$*is increasing and Schur-convex.*
- (v) *If*$\varphi$*is increasing and Schur-concave andfis decreasing and concave, then*$\psi$*is decreasing and Schur-concave.*

### **Lemma 6**

*Let the set*$\Omega \subset {\mathbb {R}}^n_+$*. The function*$\varphi :\Omega \rightarrow {\mathbb {R}}_+$*is differentiable.*

- (i) *If*$\varphi$*is increasing and Schur-convex, then*$\varphi$*is Schur-geometrically convex.*
- (ii) *If*$\varphi$*is decreasing and Schur-concave, then*$\varphi$*is Schur-geometrically concave.*

### *Proof*

We only give the proof of Lemma 6 (*i*) in detail. Similar argument leads to the proof of Lemma 6 (*ii*).

For $\varvec{x}\in I\subset {\mathbb {R}}_+$ and $x_1 \ne x_2$, we have

$$\begin{aligned}&\Delta =\left( \log x_1 - \log x_2 \right) \left( x_1\frac{\partial \varphi }{\partial x_1} - x_2\frac{\partial \varphi }{\partial x_2}\right) \\&\ \ = \left( \log x_1 -\log x_2 \right) \left( x_1\frac{\partial \varphi }{\partial x_1} - x_1\frac{\partial \varphi }{\partial x_2}+ x_1\frac{\partial \varphi }{\partial x_2} - x_2\frac{\partial \varphi }{\partial x_2}\right) \\&\ \ =x_1\frac{\log x_1 -\log x_2}{x_1 - x_2 }\left( x_1 - x_2 \right) \left( \frac{\partial \varphi }{\partial x_1} - \frac{\partial \varphi }{\partial x_2}\right) +\frac{\partial \varphi }{\partial x_2}\left( x_1 - x_2 \right) \left( \log x_1 -\log x_2 \right) . \end{aligned}$$

Since $\varphi$ is Schur-convex on $\Omega$, by Lemma 1, we have

$$\begin{aligned} \left( x_1 - x_2 \right) \left( \frac{\partial \varphi }{\partial x_1} - \frac{\partial \varphi }{\partial x_2}\right) \ge 0. \end{aligned}$$

Notice that $\varphi$ and $y=\log x$ is increasing, we have $\frac{\partial \varphi }{\partial x_2}\ge 0$, $\frac{\log x_1 -\log x_2}{x_1 - x_2 }\ge 0$ and $\left( x_1 - x_2 \right) \left( \log x_1 -\log x_2 \right) \ge 0$, so that $\Delta \ge 0$, by Lemma 2, it follows that $\varphi$ is Schur-geometrically convex on $\Omega$. $\square$

### **Lemma 7**

*Let the set*$\Omega \subset {\mathbb {R}}^n_+$*. The function*$\varphi :\Omega \rightarrow {\mathbb {R}}_+$*is differentiable.*

- (i) *If*$\varphi$*is increasing and Schur-convex, then*$\varphi$*is Schur-harmonically convex.*
- (ii) *If*$\varphi$*is decreasing and Schur-concave, then*$\varphi$*is Schur-harmonically concave.*

### *Proof*

We only give the proof of Lemma 7 (*ii*) in detail. Similar argument leads to the proof of Lemma 7 (*i*).

For $\varvec{x}\in I\subset {\mathbb {R}}_+$ and $x_1 \ne x_2$, we have

$$\begin{aligned}&\Lambda =\left( x_1 - x_2 \right) \left( x^2_1\frac{\partial \varphi }{\partial x_1} - x^2_2\frac{\partial \varphi }{\partial x_2}\right) \\&\ \ = \left( x_1 -x_2 \right) \left( x^2_1\frac{\partial \varphi }{\partial x_1} - x^2_1\frac{\partial \varphi }{\partial x_2}+ x^2_1\frac{\partial \varphi }{\partial x_2} - x^2_2\frac{\partial \varphi }{\partial x_2}\right) \\&\ \ =x^2_1\left( x_1 - x_2 \right) \left( \frac{\partial \varphi }{\partial x_1} - \frac{\partial \varphi }{\partial x_2}\right) +\frac{\partial \varphi }{\partial x_2}\left( x_1 - x_2 \right) \left( x^2_1 - x^2_2 \right) . \end{aligned}$$

Since $\varphi$ is Schur-concave on $\Omega$, by Lemma 1, we have

$$\begin{aligned} \left( x_1 - x_2 \right) \left( \frac{\partial \varphi }{\partial x_1} - \frac{\partial \varphi }{\partial x_2}\right) \le 0. \end{aligned}$$

Notice that $\varphi$ is decreasing and $y=x^2(x>0)$ is increasing, we have $\frac{\partial \varphi }{\partial x_2}\le 0$ and $\left( x_1 - x_2 \right) \left( x^2_1 - x^2_2 \right) \ge 0$, so that $\Lambda \le 0$, by Lemma 3, it follows that $\varphi$ is Schur-harmonically concave on $\Omega$.$\square$

## Simple proof of theorems

### *Proof of Theorem A*

Let $g(t)= \frac{t}{1-t}$. Directly calculating yields $g'(t)= \frac{1}{(1-t)^2}$ and $g''(t)= \frac{2}{(1-t)^3}$, it is to see that *g* is increasing and convex on (0, 1) and *g* is increasing and concave on $(1,+\infty )$.

Since $$c_n(\varvec{x},r)$$ is increasing and Schur-convex in $${\mathbb {R}}^n_+$$, from Lemma 5 (*i*) it follows that $$F_n(\varvec{x},r)$$ is increasing and Schur-convex in $$(0, 1)^n$$, and then by continuity of $$F_n(\varvec{x},r)$$ on $$[0, 1)^n$$, it follows that $$F_n(\varvec{x},r)$$ is increasing and Schur-convex on $$[0, 1)^n$$.

If *r* is even integer, then from Lemma 4, we known that $c_n(\varvec{x},r)$ is decreasing and Schur-convex, moreover *g* is increasing and concave on $(1,+\infty )$. By Lemma 5 (*ii*), it follows that $F_n(\varvec{x},r)$ is decreasing and Schur-convex.

If *r* is odd integer, then from Lemma 4, we known that $c_n(\varvec{x},r)$ is increasing and Schur-concave, moreover *g* is increasing and concave on $(1,+\infty )$. By Lemma 5 (*iii*), it follows that $F_n(\varvec{x},r)$ is increasing and Schur-concave.

The proof of Theorem A is completed. $\square$

### *Proof of Theorem B*

From Theorem A (*i*) and Lemma 6 (*i*), it follows that Theorem B (*i*) holds.

Considing

6 $$\begin{aligned} F_n(e^{\varvec{x}},r)=\sum \limits _{i_1+ i_2+\cdots +i_n=r}\left( \frac{e^{x_1}}{1-e^{ x_1}}\right) ^{i_1}\left( \frac{e^{x_2}}{1-e^{ x_2}}\right) ^{i_2}\cdots \left( \frac{e^{x_n}}{1-e^{x_n} }\right) ^{i_n}. \end{aligned}$$

Let $h(t)= \frac{e^t}{1-e^t}$. Then $h<0$ on $(0,+\infty )$. Directly calculating yields $h'(t)= \frac{e^t}{(1-e^t)^2}$ and $h''(t)= \frac{e^{t}(1+e^t)}{(1-e^t)^3}$, it is to see that *h* is increasing and concave on $(0,+\infty )$. From Lemma 4 and Lemma 5 (*ii*) (or (*iii*), respectively), it follows that if *r* is even integer (or odd integer, respectively), then $F_n(e^{\varvec{x}},r)$ is Schur-convex (or Schur-concave, respectively) on $(0,+\infty )$. And then, by Proposition 1, Theorem B (*ii*) holds.

The proof of Theorem B is completed. $\square$

### *Proof of Theorem C*

From Theorem A (*i*) and Lemma 7 (*i*), it follows that Theorem C (*i*) holds.

Considing

7 $$\begin{aligned} F_n\left( \frac{1}{\varvec{x}},r\right) =\sum \limits _{i_1+ i_2+\cdots +i_n=r}\left( \frac{1}{x_1-1}\right) ^{i_1}\left( \frac{1}{x_2-1}\right) ^{i_2}\cdots \left( \frac{1}{x_n-1 }\right) ^{i_n}. \end{aligned}$$

Let $p(t)= \frac{1}{t-1}$. Then $p<0$ on (0, 1). Directly calculating yields $p'(t)= -\frac{1}{(t-1)^2}$ and $p''(t)= \frac{2}{(t-1)^3}$, it is to see that *p* is decreasing and concave on (0, 1). From Lemma 4 and Lemma 5 (*iv*) (or (*v*), respectively), it follows that if *r* is even integer (or odd integer, respectively), then $F_n\left( \frac{1}{\varvec{x}},r\right)$ is Schur-convex (or Schur-concave, respectively) on (0, 1). And then, by Proposition 2, Theorem C (*ii*) holds.

The proof of Theorem C is completed. $\square$

## Conclusions

In this paper, using the properties of Schur-convex function, Schur-geometrically convex function and Schur-harmonically convex function, we provide much simpler proofs of Theorem A, B, C.
